# NGS in argininosuccinic aciduria detects a mutation (D145G) which drives alternative splicing of *ASL*: a case report study

**DOI:** 10.1186/s12881-016-0273-7

**Published:** 2016-02-03

**Authors:** Wei Wen, Dan Yin, Fangfang Huang, Meng Guo, Tian Tian, Hui Zhu, Yun Yang

**Affiliations:** Neonatal Screening Centre, Shenzhen Maternity and Child Healthcare Hospital, No. 3012, Fuqiang Road, Futian District, Shenzhen, Guangdong Province China; BGI-Wuhan, Wuhan, 430075 China; BGI-Shenzhen, Shenzhen, 518083 China; Department of Obstetrics and Gynecology, The Second Affiliated Hospital of Zhengzhou University, Zhengzhou, 450052 China

**Keywords:** Argininosuccinic aciduria, Next generation sequencing, Exon trapping, Alternative splicing, Molecular diagnosis

## Abstract

**Background:**

Argininosuccinic aciduria (ASAuria; OMIM 207900) is a rare autosomal recessive heterogeneous urea cycle disorder, which leads to the accumulation of argininosuccinic acid in the blood and urine. We aimed to perform genetic test to the patient and help clinician to diagnose precisely.

**Case presentation:**

In this study, we use next generation sequencing (NGS) and exon trapping to analysis the family members. We identified compound heterozygous mutations of the argininosuccinate lyase (*ASL*) gene in a Chinese Han ASAuria patient. The c.434A>G (p.(D145G)) mutation in exon 5 was shown by exon trapping to select for the formation of an alternative transcript deleted for exon 5. The c.1366C>T (p.(R456W)) mutation had been previously reported in an Italian patient.

**Conclusions:**

This is the first report of a missense mutation driving alternative splicing which results in the loss of exon 5 in ASAuria. This study also demonstrates the value of NGS in the identification of mutations and molecular diagnosis for ASAuria families.

**Electronic supplementary material:**

The online version of this article (doi:10.1186/s12881-016-0273-7) contains supplementary material, which is available to authorized users.

## Background

Argininosuccinic aciduria (ASAuria; OMIM 207900) is an inborn error of metabolism affecting the urea cycle. It is caused by mutations in the argininosuccinate lyase (*ASL*) gene, the protein product of which cleaves argininosuccinate to fumarate and arginine. The prevalence of ASAuria is 1 in 70,000 live births making it the second most common urea cycle disorder [1].

Urea cycle disorders result from defects in the metabolism of waste nitrogenous compounds derived from the breakdown of proteins and other nitrogen-containing molecules. Patients accumulate the nitrogen in the form of ammonia in plasma, which is a highly toxic substance that is not excreted. Six genetic forms have been identified, arising from inherited deficiencies of catalytic enzymes (CPS1, OTC, ASS1, ASL, ARG1) and a cofactor-producing enzyme (NAGS). With the single exception of OTC which is X-linked, all show autosomal recessive inheritance [2].

The clinical presentation of patients with ASAuria is variable. Generally, the disease has two forms, a severe neonatal form and a milder late onset form [3]. The severe neonatal form is characterized by hyperammonemia within the first few days of life with poor feeding, vomiting, lethargy, and seizures, with subsequent progression to coma. The late onset form manifests late in infancy or in childhood; it presents with mental retardation, vomiting, failure to thrive and behavioral problems [4].

The clinical diagnosis is confirmed by measuring ammonia and argininosuccinate levels in plasma [5]. The human *ASL* gene spans approximately 17 kb (RefSeq NG_009288.1) and comprises 16 exons (RefSeq NM_001024943.1 coding region starts at exon 1), located on chromosome 7cen-q11.2 [6]. Previous studies have reported alternatively spliced transcripts of ASL in all investigated cells and tissues, mainly involving deletions of exon 2 and 7 [7–9].

A customized capture array (NimbleGen, Roche, USA) was designed to capture genes including the six recognized urea cycle disorder genes plus *SLC25A13* and *SLC25A15*, encoding citrin and mitochondrial ornithine transporter, respectively, both of which play important roles in the urea cycle. Until now, there are 39 unique DNA variants reported by LOVD (http://chromium.lovd.nl/LOVD2/home.php?select_db=ASL) and the number of reported mutations is still quite small. Our study aimed to identify causative mutations by NGS on a Chinese pedigree with ASAuria and to analyze the underlying molecular defects.

## Case presentation

The patient (2.1), a Chinese Han boy, was brought to the Shenzhen Maternal and Child Healthcare Hospital at 6 days of age with jaundice, poor feeding, vomiting but without crying. At 7 days of age, he was admitted to the hospital with presumed neonatal sepsis, neonatal conjunctivitis and neonatal jaundice. He was lethargic with poor responses and had ocular hyperemia with purulent secretions from eyes. The patient was kept NPO for several hours. Intravenous dextrose containing solution was given and he was treated with antibiotics for presumed sepsis. After one day at the hospital, his condition became worse with hypotonia and coma. Biochemical analysis revealed hyperammonemia (456 μmol/L, normal range 18–72 μmol/L) in the serum, which persisted on the following days (568 μmol/L). Tandem Mass Spectrometry revealed the serum amino acids levels : citrulline 127.2 μmol/L (4–24 μmol/L), phenylalanine 23.1 μmol/L (25–120 μmol/L), tyrosine 13.99 μmol/L (25–150 μmol/L), valine 68.6 μmol/L (80–250 μmol/L), He was treated with amikacin and arginine to reduce the level of serum ammonia, after 3 days, the serum ammonia decreased to 51 μmol/L, on the ninth day after admission to the hospital, the level of serum ammonia was 21 μmol/L and he was sent out of the hospital.

**Fig. 1:**
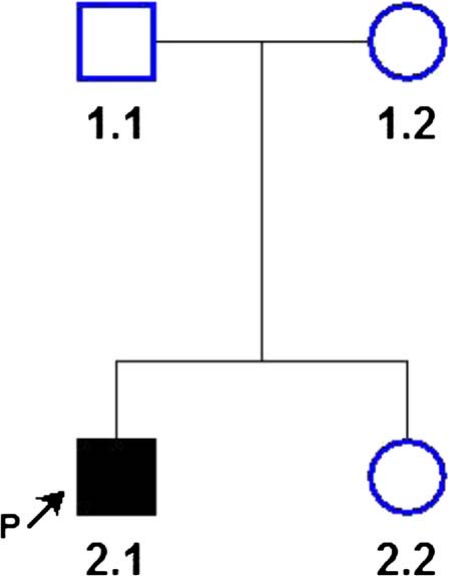
ASL genotypes, determined by NGS using a urea cycle disorders chip, are indicated. Pedigree showing autosomal recessive inheritance of argininosuccinic aciduria. Solid symbol, affected; open symbol, unaffected; arrow, proband (P)

After leaving the hospital, he was followed regularly and treated with low-protein diet, oral arginine tablets, sodium benzoate. His daily protein intake was restricted to ≤1.5 g/Kg per day and serum ammonia was maintained at ≤80 μmol/L. At 4 years of age, the patient presented acceptable physical development, but his language and motor development slightly behind his peers’ children. He had a six year old sister who presented a normal condition. The pedigree is shown in Fig. 1.

Following informed consent, the patient, his parents and older sister’s peripheral blood (3 ml) were collected and anticoagulated with EDTA. Total DNA was extracted using the QIAamp DNA extraction kit (Qiagen, Hilden, Germany), according to the manufacturer’s protocol.

DNA samples were sequenced using Microarray-based NGS. We designed a custom array from Roche NimbleGen (Madison, USA) to capture exons (including the 10 bp flanking either side of each) of genes including 8 genes associated with urea cycle disorders. Genomic DNA was fragmented into fragments ranging from 200–300 bp using an ultrasonoscope (Covaris S2, Massachusetts, USA). Primers and adapters were then ligated to the purified DNA fragments to construct the library. The library was amplified by PCR and hybridized to the capture array. Samples were sequenced on Illumina HiSeq2500 Analyzers (Illumina, San Diego, USA) for 90 cycles to generate 90 bp paired-end reads. Image analysis and base calling were performed using the Illumina Pipeline [10].

Exon 5 and exon 16 of the ASL gene were amplified using 200 ng of genomic DNA, 1 μM each of primers (*ASL*-exon5-F-GGCTCCTCAGGGAAGCAACA, *ASL*-exon5-R-AGTTCTGGGATGCCCCTGTC, *ASL*-exon16-F-AAGTGAGCCTGGGTGCCTGG, *ASL*-exon16-R-CGAAAGCCCAGCAACGAGG), 0.25 mM dNTPs and 1U Taq polymerase in 1 × buffer with annealing temperature at 64 and 67° separately. PCR products were purified using the QIA PCR purification kit (Qiagen, Crawley, UK).

To study the function of the c.434A>G (p.(D145G)) mutation in exon 5, we performed exon trapping studies in vitro [11]. We amplified a genomic fragments containing exon 5, intron 5 and exon 6 with primers F-CCGTGTTGTCCCAACCTTGA and R-GGGCTGTGCTAGAGGGGA from the patient and from a normal individual, respectively. The product fragments were cloned into the pSPL3exon trapping vector(Invitrogen, Carlsbad, CA). Wild type (WT) and mutant plasmids were then transfected into the COS7 cell line using Lipofectamine2000 (Invitrogen, Carlsbad, CA) respectively. After culture for 48 hours, total RNA was extracted using Trizol (TaKaRa, Dalian, China). 5 μg RNA was reverse transcribed to cDNA in a total volume of 20 μl with superscript II RNAse H-reverse transcriptase and oligo-dT priming (TaKaRa, Dalian, China). The cDNA was amplified using vector primersSD6(TCTGAGTCACCTGGACAACC) and SA2 (ATCTCAGTGGTATTTGTGAGC), PCR products were separated on a 2 % TBE agarose gel. After purification, amplification products were characterized by direct sequencing.

**Fig. 2:**
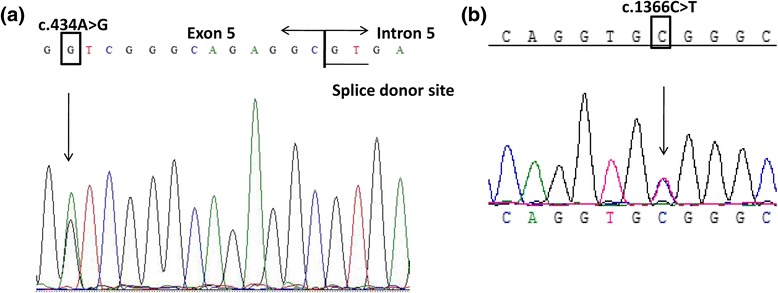
Direct sequencing of ASL (NCBI Reference Sequence: NM_001024943.1) in the patient 2.1 (other family members’ data not shown). **a** The heterozygous mutation c.434A>G (p.(D145G)) in exon 5 (arrow). **b** The heterozygous mutation c.1366C>T (p.(R456W)) in exon 16 (arrow)

NGS identified ten variants in total, located in four different urea cycle related genes in the patient (Additional file 1: Table S1). These included compound heterozygous mutations in *ASL*, of which one was a mutation: c.434A>G (p.(D145G)) located in exon 5; the other c.1366C>T mutation in exon 16 had been reported previously [4]. The patient’s father and sister were heterozygous for c.434A>G and his mother was heterozygous for c.1366C>T. We validated the results of the NGS by direct sequencing (Fig. 2a-b).

**Fig. 3:**
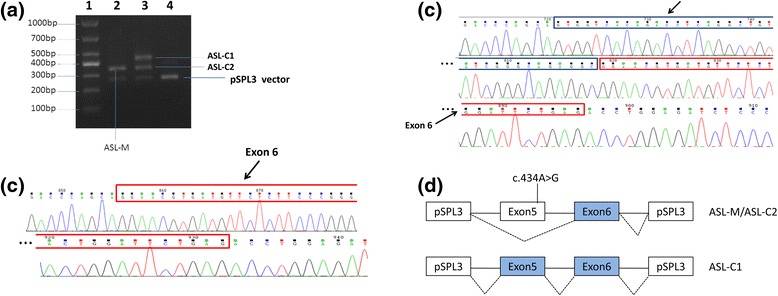
Functional analysis of the mutation c.434A>G in ASL and its effect on mRNA splicing. **a** cDNA gel electrophoresis: 1, DNA molecular weight marker (100–1000 bp); 2, c.434A>G mutated cDNA; 3, wild type cDNA; 4, pSPL3 plasmid control. **b** Sequence of the mutated cDNA amplification product ASL-M and wild type cDNA amplification product ASL-C2, both showing exon 5 deletion. The red box represents exon 6. **c** Sequence of wild type cDNA amplification product ASL-C1 showing the reference sequence. The dark blue box represents exon 5, red box represents exon 6. **d** Scheme for the effects of the mutation c.434A>G. ASL-M is exactly the same as ASL-C2 with splicing out of exon 5. The sequence of ASL-C1 identical to the reference sequence, without alternative splicing

We performed an in vitro exon trapping assay to analyze the effects of c.434A>G upon splicing of the transcript. The gel electrophoresis of the cDNA products is shown in Fig. 3a. Lane2 contains cDNA products trapped from the c.434A>G mutant construct, showing a single band of ~350 bp (ASL-M). Lane 3 contains cDNA products trapped from the wild type control construct, showing two bands of ASL-C1 (~450 bp) and ASL-C2 (~350 bp). Direct sequencing showed that the sequence of ASL-M was exactly the same as ASL-C2, with alternative splicing resulting in the deletion of exon 5 (Fig. 3b). The sequence of ASL-C1 was identical to the reference sequence, with no alternative splicing (Fig. 3c). We drew a schematic diagram to describe the mutation c.434A>G led to the skipping of exon 5 (Fig. 3d). Functional analysis of this mutation demonstrates that the substitution of nucleotide (c.434A>G) leads to the complete loss of exon 5.

## Discussion

In this study, we identified compound heterozygous mutations in *ASL* using NGS, confirming the clinical diagnosis of ASAuria. We developed an in vitro exon trapping assay which demonstrated that the mutation c.434A>G completely abolished normal splicing of exon 5. At the beginning, We have amplified the cDNA reverse-transcripted from the patient’s mutant mRNA, however because of high numbers of ASL transcripts (10 transcripts), which makes RT-PCR is not suitable for analyzing alternative splicing of the transcript. Furthermore, the quantity of RNA is hard to extract enough from patient. While additional factors could potentially influence the splicing pattern in vivo, the restricted availability of patient samples makes the exon trapping assay a useful tool for mutation assessment. Also, there is some other physiological skipping of various ASL exons than 2 and 7 detected by Linnebank and colleagues (2000).

A previous report described the other mutation, c.1366C>T (p.(R456W)), which involves a conserved arginine in the terminal alpha helix of the protein. Substitution with tryptophan is predicted to cause a displacement and to shift the position of glutamine454 [4]. Our data suggest that compound heterozygosity for these two mutations is unlikely to result in translation of fully functional ASL protein.

The molecular diagnosis of the urea cycle disorders is an important area for development. Although determination of ASL activity in cultured fibroblasts or erythrocytes is a reliable method to confirm the diagnosis, it requires the availability of patient samples and is a complex method only available in a few laboratories worldwide. However, molecular analysis is more feasible and potentially efficient. Therefore, we recommend NGS technologies to diagnosis ASAuria and other urea cycle disorders.

Overall, this is the first report of a pathogenic missense mutation causing alternative splicing which results the loss of exon 5 in ASAuria. It helps us understand the molecular mechanism of ASL. This study also demonstrates the value of NGS in the identification of mutations and molecular diagnosis in these families.

## Conclusions

In conclusion, we identified compound heterozygous mutations in *ASL* using NGS, confirming the clinical diagnosis of ASAuria. The c.434A>G (p.(D145G)) mutation in exon 5 was shown by exon trapping to select for the formation of an alternative transcript deleted for exon 5. This is the first report of a missense mutation driving alternative splicing which results in the loss of exon 5 in ASAuria.

### Consent to publish

Written informed consent was obtained from the patient’s parents for publication of this case report and any accompanying images. A copy of the written consent is available for review by the Editor of this journal.

### Consent to participate

Patient’s parents agreed their son (the patient) and daughter to take part in the study. Blood sample collection conforms to the routine standard care.

### Ethics approval

The research was prospectively reviewed and approved by a duly constituted ethics committee (The Institutional Review Board on Bioethics and Biosafety of Beijing Genomics Institute Ethical Approval).

## Additional file

Additional file 1: Table S1. 10 Variants identified in 8 urea cycle related genes by targeted array NGS. (XLSX 10 kb)

## Abbreviations

ASAuria: argininosuccinic aciduria
ASL: argininosuccinate lyase
NGS: next generation sequencing
